# *NAC* Gene Family in *Lagerstroemia indica*: Genome-Wide Identification, Characterization, Expression Analysis, and Key Regulators Involved in Anthocyanin Biosynthesis

**DOI:** 10.3390/cimb47070542

**Published:** 2025-07-11

**Authors:** Zilong Gao, Zhuomei Chen, Jinfeng Wang, Weixin Liu

**Affiliations:** 1 Zhejiang Academy of Forestry, Hanzhou 310023, China; gaozillong@163.com (Z.G.); zhuomeichen@163.com (Z.C.); 2 Research Institute of Subtropical Forestry, Chinese Academy of Forestry, Hangzhou 311400, China

**Keywords:** *Lagerstroemia indica*, NAC, anthocyanin, gene expression

## Abstract

NAC (NAM, ATAF1/2, CUC1/2) is a plant-specific transcription factor (TF) family that plays important roles in various physiological and biochemical processes of plants. However, the *NAC* gene family in *Lagerstroemia indica* and its role in anthocyanin metabolism are still unexplored. In our study, a total of 167 *NACs* were identified in the *L. indica* genome via genome-wide analysis and bioinformatics techniques. Amino acid sequence analysis showed that all 167 NAC proteins contained a conserved NAM domain. This domain primarily comprised random coils, extended strands, and alpha helices. Most *NACs* were found on the nucleus and dispersed over 23 of the 24 plant chromosomes. Based on phylogenetic analysis, the *NACs* can be categorized into ten subgroups. Furthermore, the promoter homeotropic elements predicted the cis-acting elements in the promoters of these genes related to hormones, development, environmental stress response, and other related responses, demonstrating the diverse regulatory mechanisms underlying gene functions. In addition, a co-expression network was established through RNA sequencing. This network helped identify seven key *LiNACs*, genes related to anthocyanin expression (CHS) and transcription factors (MYB and bHLH). To identify potential anthocyanin regulatory factors present in *L. indica* petals, protein interaction prediction was performed, which revealed that LiNACs might participate in anthocyanin regulation by interacting with other proteins, such as MYB, ABF, ABI, bZIP, MYC, etc. Our results provided novel insights and could help in the functional identification of *LiNACs* in *L. indica* and the regulation of anthocyanin synthesis.

## 1. Introduction

The regulation of expression of transcription factors (TFs) is crucial in plants, as it impacts plant growth, development, and pigment expression by regulating the expression of target genes [1]. TFs regulate the expression of target genes by binding to specific cis-acting promoter elements [2]. In plants, more than 50 different TF families have been identified via bioinformatics analysis [3].

The NAC (NAM, ATAF1/2, CUC1/2) protein family is one of the largest plant-specific TF families. For instance, 105, 140, 101, 45, and 163 *NACs* have been identified in Arabidopsis, rice [4], soybean [5], *Camellia sinensis* [6], and *Populus trichocarpa*, respectively [7]. NAC proteins typically have a conserved NAM domain at the N-terminus. This domain comprises about 160 amino acid residues and is divided into five subdomains (A–E) [8]. The structural NAM domain harbors a region with 60 residues containing a unique TF fold, composed of twisted fragments defined by several helical elements [9].

*NAC* genes are involved in a wide range of plant development processes, including the development of plant stem apical meristematic tissues [10], floral morphogenesis [11], lateral root development [12], leaf senescence [13], flowering induction [14], embryonic development [9], cell cycle control [15], hormone signaling, etc. [16]. Additionally, studies have shown that *NACs* in some plants are involved in regulating the biosynthetic pathways of anthocyanins. For instance, CmNAC25 in *Chrysanthemum morifolium* positively regulates anthocyanin accumulation in the petals of chrysanthemums during the late flowering stage by regulating CmMYB6 [17]. When lychee fruit matures, LcNAC002 binds to the promoters of the key genes *LcSGR* (for chlorophyll degradation) and *LcMYBY1* (for anthocyanin synthesis) and activates them, thereby regulating the color of lychee peel [18]. LcNAC13 binds to the promoter of anthocyanin synthesis-related genes and inhibits their transcription [19]. *PpNAC25* overexpression in peach upregulates anthocyanin biosynthesis and transports genes at the transcriptional level, increasing anthocyanin content [20].

*Lagerstroemia indica*, a deciduous small tree or shrub of the Lythraceae family, is often planted in tropical and warm temperate regions, with its flowering period mainly in summer [21]. As a traditional plant in China, it is often cultivated for horticulture, urban greening, and medicinal purposes [22]. Research on *L. indica* has shown that it exhibits pharmacological effects, such as anti-inflammatory, analgesic, antipyretic [23], antioxidant [24], antibacterial, anti-cancer, anti-diabetic, etc. [25]. Due to the wide variety of colors in *L. indica*, including white, red, and pink petals, it is often used as an ornamental plant [26,27]. Anthocyanins, important plant pigments, play an important role in the regulation of *L. indica* color. However, the regulatory mechanism underlying anthocyanin synthesis in *L. indica* needs further exploration.

We comprehensively characterized the *NAC* family genes in *L. indica* to reveal the potential regulatory factors related to anthocyanin synthesis. Subsequently, we performed sequence characterization, phylogenetic analysis, three-dimensional protein structure construction, and chromosome localization. Further, we analyzed the expression patterns of *NACs* underlying different flower colors. Based on co-expression network analysis, we identified seven *LiNAC* genes and TFs associated with anthocyanin synthesis. Through the prediction of promoter homeoregulatory elements and protein interaction networks, we found that these genes might play important roles in flower color regulation.

## 2. Materials and Methods

### 2.1. Plant Materials

This plant material grows in the Lagerstroemia Germplasm Resources of the Zhejiang Academy of Forestry in Hangzhou, Zhejiang Province. The selected variety of *Lagerstroemia indica* is a semi hybrid offspring of ‘Jianmin Hong’. This study selected three color varieties—white (W), red (R), and purple (P). All plants were approximately 10 years old and had received regular irrigation and fertilization. Sampling was carried out in the summer of 2023, with blooming petals randomly collected from healthy plants. Each flower’s color is sampled three times (with no less than 5 g per sample), and the petals are stored in liquid nitrogen at −80 °C until subsequent transcriptome studies and Quantitative Real-Time PCR (qRT-PCR) experiments are conducted.

### 2.2. Sequence Analysis of TFs in LiNAC

The amino acid sequence of *L. indica* was sourced from the China National Center for Bioinformatics (PRJCA013427). NAC TFs in *L. indica* were identified using BLASTP and Hmmer search methods. Using the NAC DNA binding domain HMM (hidden Markov model) profile (pfam Number: PF01849) as a reference, candidate NAC TFs were identified through HMMER3.4 (Eddy/Rivas, Cambridge, MA, USA). The HMM configuration file was downloaded from the Pfam database (https://pfam.xfam.org/, accessed on 5 January 2025), and the longest amino acid sequence in each gene was selected. Moreover, we sorted and collected NAC TFs from *Arabidopsis thaliana*, rice [4], tea tree [6], and *Populus tomentosa* [7] by conducting BLSTP gene screening with the coding sequence (CDS) of the *L. indica* genome and comparing it with HmmerSearch results. The TFs at the intersection of the two sets of results were selected and assessed using SMART (http://smart.embl-heidelberg.de/, accessed on 12 January 2025) to verify whether they contained NAC TF features.

### 2.3. Sequence Analysis of L. indica NACs

WoLF PSORT (https://wolfpsort.hgc.jp/, accessed on 13 January 2025) was used for subcellular localization prediction. Gene structure and collinearity analysis of *LiNAC* genes between species (*L. indica* and *Arabidopsis*) was analyzed using TBtools (v.2.135) (South China Agricultural University, Guangzhou, China). The coding region structure analysis was visualized based on the annotation file (GFF) of the *Lagerstroemia* genome. Using the MEME online website (https://meme-suite.org/meme/, accessed on 15 January 2025), the conserved motif sequences of NAC TFs were predicted in *L. indica*. NACBI Batch CD (https://www.ncbi.nlm.nih.gov/Structure/bwrpsb/bwrpsb.cgi, accessed on 15 January 2025) search was conducted for the structural domains of NAC TFs in *L. indica*, which were then visualized using TBtools (v.2.135). We used MEGA7 software (Mega Limited, Auckland, New Zealand) to analyze the structural domain characteristics of repeat sequences in 167 *NACs* in *L. indica* and the TBtools software (South China Agricultural University, Guangzhou, China) to draw the gene logo of the conserved structural NAM domain. NPS@ (https://npsa.lyon.inserm.fr/cgi-bin/npsa_automat.pl?page=/NPSA/npsa_server.html, accessed on 15 January 2025) and SWISS-MODEL (https://swissmodel.expasy.org/, accessed on 15 January 2025) were used to construct the secondary structure and 3D model of NAM. plantCARE (https://bioinformatics.psb.ugent.be/webtools/plantcare/html/, accessed on 17 January 2025) was used to investigate the cis-acting elements in the upstream region of the *NAC* TF promoters in *L. indica*. After collecting and organizing, these cis-acting elements were divided into four modules by length (0–499, 500–999, 1000–1499, and 1500–2000 kb) via chiplot (https://www.chiplot.online/, accessed on 25 January 2025) and stacked bar charts and website heat maps were created. GSDS (https://gsds.gao-lab.org/, accessed on 26 January 2025) was used to analyze the number and structure of introns and exons of 167 *NACs* in *L. indica* were analyzed. The results were processed using Adobe Photoshop 2020 software (Adobe, San Jose, CA, USA) to draw the coding region structure of *L. indica NAC*. The “Gene Location Visualize from GTF/GFF” function in TBtools (v.2.135) was used for chromosome localization analysis and visualization on 167 *NAC* TFs in *L. indica*.

### 2.4. Phylogenetic Analysis

Multiple sequence alignment of 167 full-length NAC proteins in *L. indica* was conducted using ClustalW (University College Dublin, Dublin, Ireland). The phylogenetic tree was conducted by the neighbor-joining method (NJ) with 1000 bootstrap replicates [28]. The phylogenetic tree was then beautified using ITOL (https://itol.embl.de/, accessed on 20 January 2025).

### 2.5. Weighted Correlation Network Analysis (WGCNA) and Construction of Protein Interaction Networks

The transcriptome data (SUB14762385) of petals (white, pink, and red) was selected from three different colors of the half-sibling family of the ‘Jianmin Hong’ variety of *L. indica* from the NCBI website, and WGCNA R Shiny was used to analyze gene co-expression [29]. By filtering genes with low expression changes (standard deviation ≤ 0.1) and setting the power value to 1–30, the corresponding correlation coefficients and average connectivity of the network were calculated. Based on the selected power values, a weighted co-expression network model was constructed to divide 28,367 genes into nine modules. The Grey module could not be assigned to any gene set of any module and had no reference significance. Using the Pearson correlation algorithm to calculate the correlation coefficient (*p* Value) between module feature genes and traits, with a threshold of absolute correlation coefficient ≥ 0.3 and *p* Value < 0.05, the correlation between the expression levels of anthocyanins in these nine modules and the three types of petals was calculated. Screening was performed by constructing trait module correlation heat maps and scatter plots of specific traits and module genes. Finally, we selected the most significant positive and negative correlation modules (darkred and royalblue) of *NAC*-related genes among the modules with the highest correlation with petals for further analysis.

The *L. indica NAC* gene (*LiNAC*) and its co-expressed genes (weight ≥ 0.1) selected from the darkred and royalblue modules were annotated using SwissProt (Swiss Institute of Bioinformatics, Geneva, Switzerland). The co-expression regulation network spectrum was drawn using Cytoscape (3.10.3) (University of California San Diego, La Jolla, CA, USA) based on SwissProt annotation, and their expression heatmaps were plotted using Chiplot (https://www.chiplot.online/, accessed on 22 January 2025). Using plantCARE (https://bioinformatics.psb.ugent.be/webtools/plantcare/html/, accessed on 24 January 2025), the *LiNAC* TFs and genes related to the anthocyanin synthesis path way were screened [30] and investigated for homeomorphic elements by selecting a 2-kbp upstream region of the promoter.

The *LiNACs* were selected from the co-expression network to construct the interaction network. Then, NAC proteins related to Arabidopsis species and proteins involved in anthocyanin regulation were selected from the STRING network (https://cn.string-db.org/, accessed on 2 February 2025). Enable all network evidence types, including text mining, experiments, databases, co-expression, neighborhood, gene fusion, and co-occurrence. The minimum interaction score threshold was set to 0.15, and the network data were visualized using Cytoscape (v3.10.3) (University of California, San Diego, CA, USA).

### 2.6. qRT-PCR Analysis of 7 LiNAC Genes

*LiNACs* were selected for qRT-PCR analysis. Primer Premier 6.0 software (Premier Biosoft, Palo Alto, CA, USA) was used to design the specific primers (Supplementary Table S1), and 18sRNA was used as the internal reference gene. The qRT-PCR was carried out as described previously [17].

## 3. Result

### 3.1. Identification of NACs in L. indica

Based on literature screening, AtNACs, OsNACs [4], CsNACs [5], and PtNACs [7] were selected as samples. Next, we performed BLASTP using TBtools, and Hmmer was used to search for *L. indica* NACs in the Pfam database. By comparing the *L. indica* NACs searched in BLASTP and Pfam databases, a total of 177 NACs were screened. Ten genes that were not recognized in the Pfam database were statistically removed, resulting in a total of 167 *L. indica* NAC TFs.

### 3.2. Analysis of Physical and Chemical Properties of L. indica NACs

Analysis of the physicochemical properties using TBtools showed that the molecular weights of *L. indica* NACs were in the range of 17,742.15–196,244.76 Da, with theoretical isoelectric points (PI) of 4.51–9.84. Based on the instability coefficient, 135 and 32 NACs were found to belong to unstable and stable proteins (coefficients >40 and <40), respectively. Analysis of the fatty index and hydrophilicity coefficient revealed that all 167 NAC proteins were hydrophobic (Supplementary Table S2).

### 3.3. Conserved Domain Analysis of NAC in L. indica

**Figure 1:**
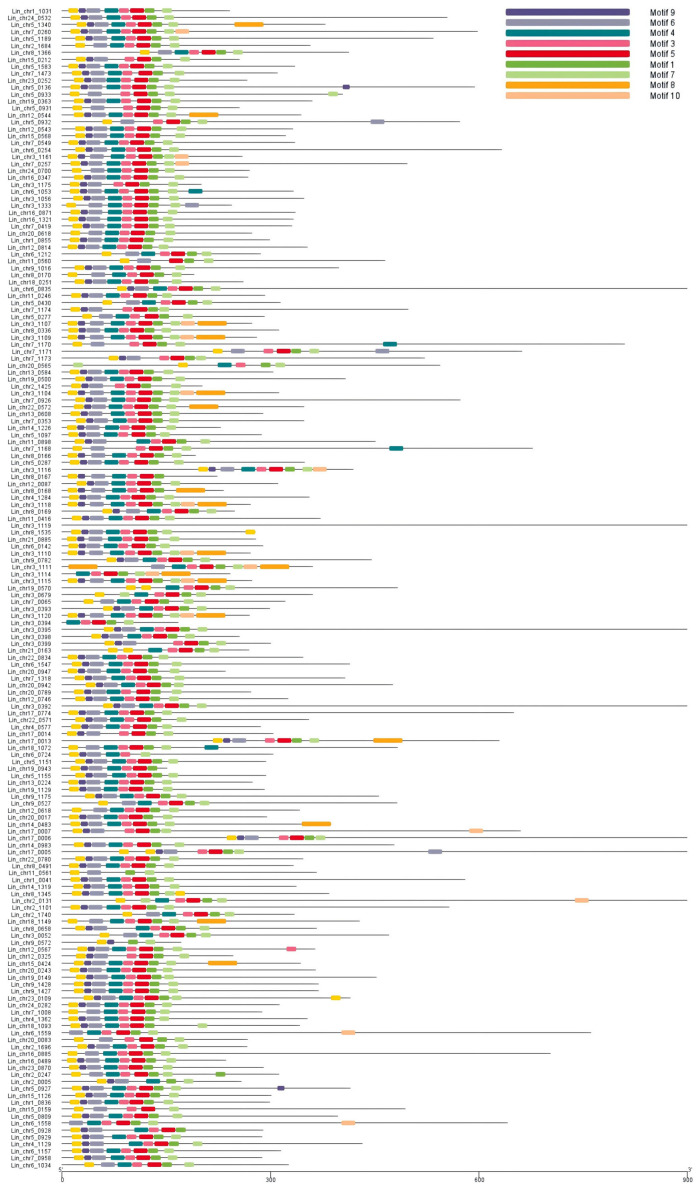
Conserved domains in 167 NACs of *Lagerstroemia indica.* Different colors represent different motifs.

**Figure 2:**
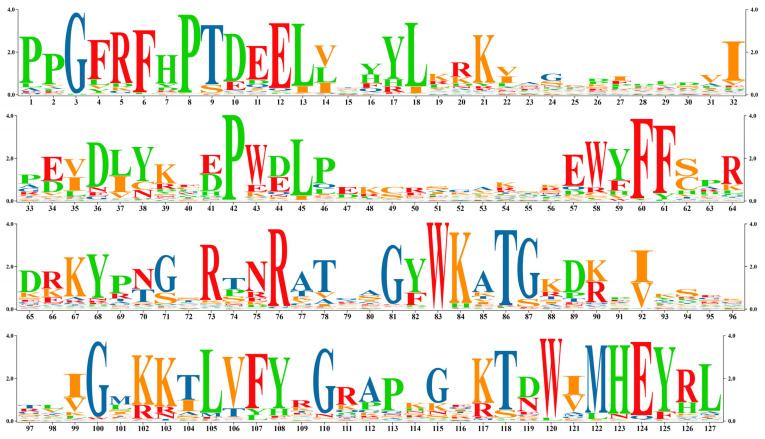
Logo of conserved structural domain genes in the NAM region. The horizontal axis represents length, and the vertical axis represents repeated values. The higher the repetition value, the stronger the homology of the region.

CD Search was used for structure analysis of the 167 NACs. Motif analysis showed that the NAM region sequences of the 167 proteins were highly conserved (Figure 1). We aligned and adjusted this conserved sequence (Figure 2) using the MEGA7 software and visualized the gene logo using the TBtools software (v.2.135).

**Figure 3:**
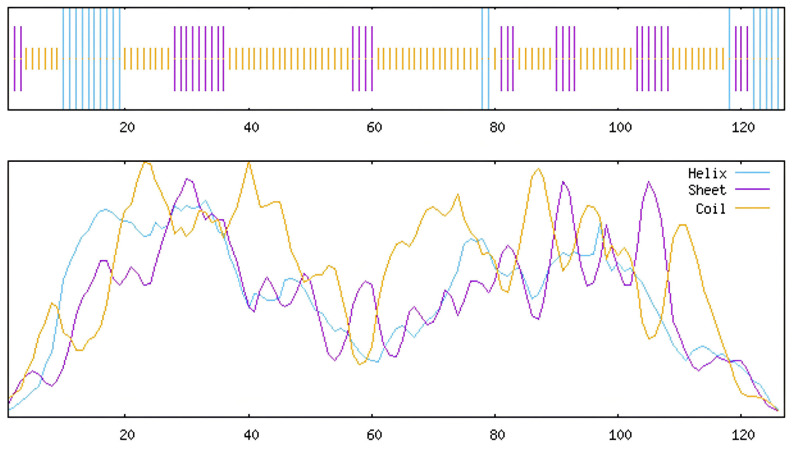
Secondary structure diagram of the conserved domain of *L. indica* NAM.

**Figure 4:**
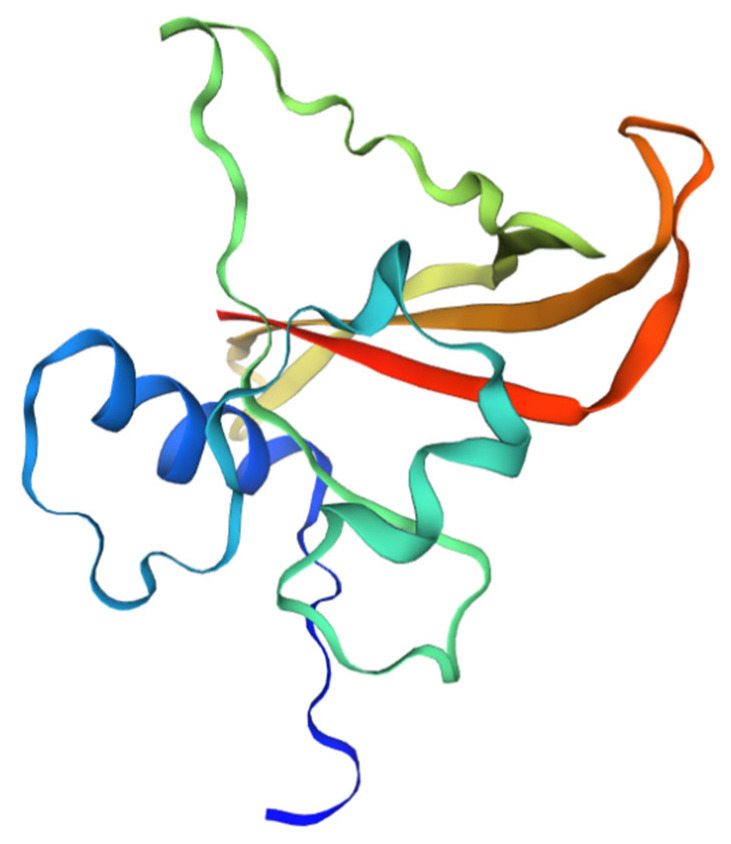
Three-dimensional model of conserved structural domain of *Lagerstroemia indica* NAM.

Analysis of the secondary structure of the conserved NAM domain using NPS@: SOPMA revealed that the secondary structures primarily included random coil (61.42%), extended strand (24.41%), and alpha helix (14.17%; Figure 3). Three-dimensional modeling using SWISS-MODEL and analysis using Seq Identity revealed that its similarity with NAC-related proteins of *Setaria italica* was as high as 94.49% (Figure 4).

### 3.4. GSDS Analysis of L. indica NAC Structure

**Figure 5:**
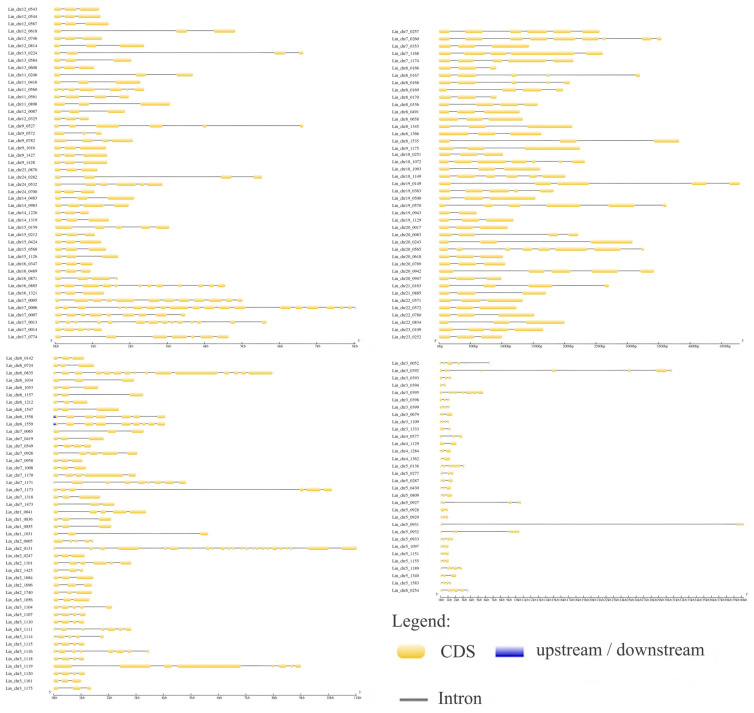
Structural analysis of *Lagerstroemia indica NACs*.

Structural analysis of 167 *LiNACs* on GSDS (Gene Structure Display Server) showed that three of these genes (1.79%) harbored two exons and one intron, 91 genes (54.49%) carried three exons and two introns, 23 genes (13.77%) had four exons and three introns, 19 genes (11.38%) had five exons and four introns, eight genes (4.79%) carried six exons and five introns, and 23 genes (13.77%) had >6 exons and >5 introns (Figure 5).

### 3.5. Prediction of Subcellular Localization of L. indica NACs

Subcellular localization of the 167 NACs was predicted using Wolf PSORT. In the process of subcellular localization prediction for these 167 NACs, we conducted segment-based localization probability analysis and assigned the highest-probability region as each protein’s definitive localization. By analyzing the localization of these genes in different parts of the cell, it was found that these genes were localized to eight locations (nucleus, cytoplasm, chloroplasts, mitochondria, plasma membrane, vacuolar membrane, cytoskeleton, and peroxisomes). Among them, 120 (72.3%), 16 (9.6%), 13 (7.8%), five (3.0%), four (2.4%), and two LiNACs (1.2%) were localized to the nucleus, cytoplasm, chloroplasts, mitochondria, plasma membrane and vacuolar membrane, and cytoskeleton and peroxisomes, respectively (Supplementary Table S3).

### 3.6. Constructing a Phylogenetic Tree of L. indica NACs

**Figure 6:**
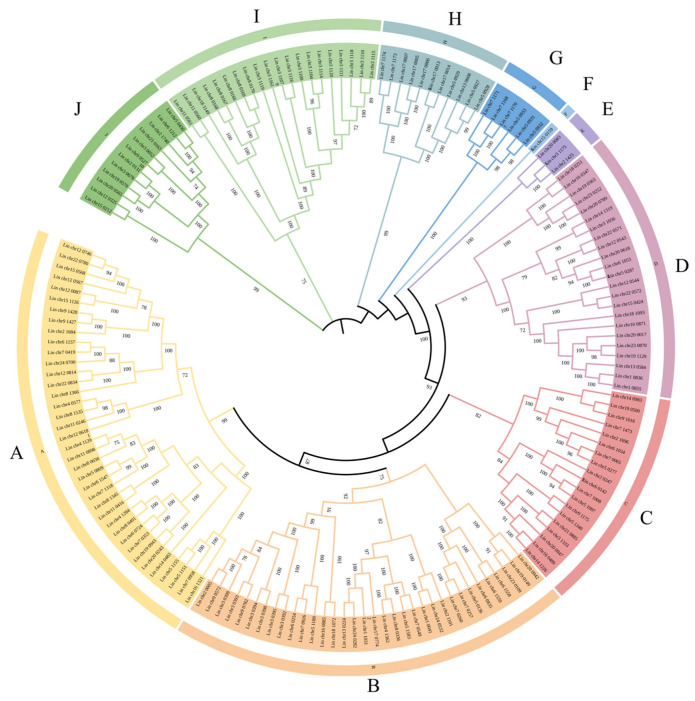
Phylogenetic tree of *Lagerstroemia indica* NAC. Different colored groups represent different subgroups, where subgroups are sorted from A to J based on their phylogenetic relationships.

Using NJ, a phylogenetic tree was constructed using MEGA7 and plotted on ITOL. By selecting the correlation coefficient (bootstrap) to 0.7, LiNACs were further subdivided into ten subgroups (A–J; Figure 6). The largest subgroup A harbored 38 LiNACs, while the smallest subgroup only had one gene (Lin_chr15_0159).

### 3.7. Chromosomal Localization and Collinearity Analysis of L. indica NACs

**Figure 7:**
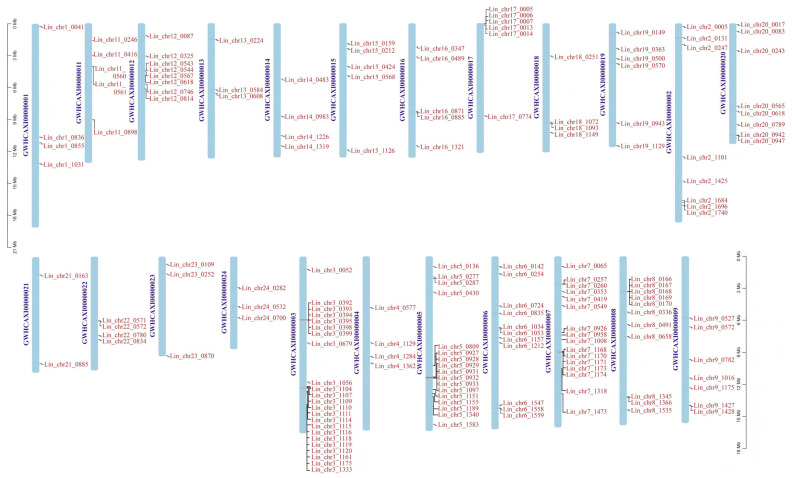
Chromosome mapping of 167 *NACs* in *Lagerstroemia indica*.

Chromosome localization analysis showed that *NACs* were distributed across 23 chromosomes in the *L. indica* genome. We found that some *NACs* were tightly arranged in certain regions of some chromosomes (Figure 7). For instance, chromosomes 3, 5, 6, 7, and 8 harbored a high number of *LiNACs* that were tightly arranged.

**Figure 8:**
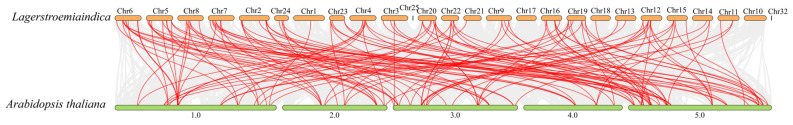
Co-temporal study of *NAC* genes on the chromosomes of *Lagerstroemia indica* and *Arabidopsis thaliana*. Different body blocks represent different chromosomes, and different colors represent different species, with orange representing *L. indica* and green representing *A. thaliana*. The red line represents the collinear relationship of “genomes” in different species.

Through homology analysis, both *Lagerstroemia indica* and *Arabidopsis thaliana* have direct homologs of the *NAC* gene. Therefore, we conducted inter species chromosomal collinearity analysis between the 167 *NAC* genes selected from *L. indica* and the 106 *NAC* genes in *A. thaliana*. The analysis results showed that there were 85 pairs between *L. indica* and *A. thaliana* (Figure 8).

### 3.8. Analysis of NAC Promoter Components in L. indica

**Figure 9:**
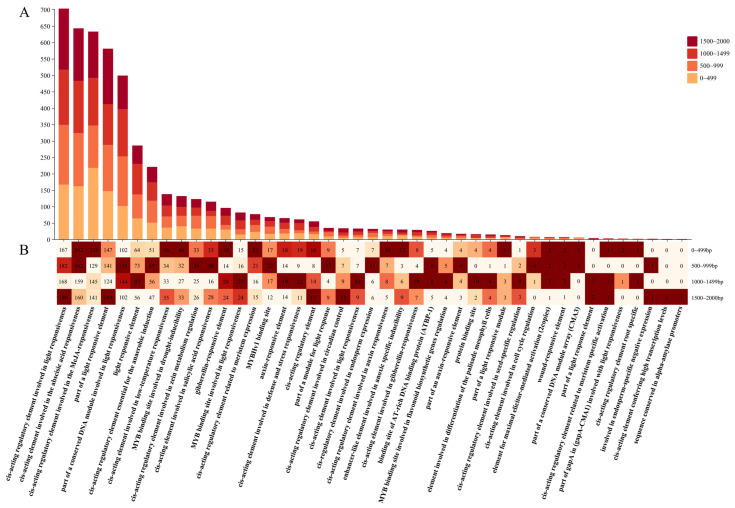
Prediction of homeotropic elements in 167 NAC proteins of *Lagerstroemia indica* ((**A**). Stacked bar chart representing the total number of cis-acting elements in each category; (**B**). statistical analysis of 43 cis-acting elements in the four regions of the promoter (0–499, 500–999, 1000–1499, and 1500–2000 bp), with the numbers in the grid indicating the number of homeotropic elements).

We used the 2 kb region upstream of the CDS of 167 *NAC* TFs in *L. indica* as the promoter region to predict the cis-acting elements. By counting the number of these homeotropic elements, a total of 43 types of ecosystem elements were discovered (Figure 9A). Among them, five types of functional elements, namely cis-active regulatory elements involved in light, abscisic acid (ABA), and methyl jasmonate (MeJA) responsiveness, part of a light responsive element, and part of a conserved DNA module involved in light responsiveness, account for the highest proportion of the elements (61.6%). In addition, we found MYB binding sites in these promoters, indicating they were involved in the regulation of flavonoid biosynthesis genes, light response, and drought response. To understand the regulatory function of the *LiNACs*, we divided the promoter region into four parts (0–499, 500–999, 1000–1499, and 1500–2000 bp). Furthermore, we found that the cis-active regulatory element involved in light responsiveness was mostly widely distributed across all regions. Most elements related to environmental response and meristematic expression, plant growth and development, and light response were distributed in the ranges of 0–499, 500–999, and 1000–2000 bp, respectively (Figure 9B).

### 3.9. Expression Analysis of LiNACs

**Figure 10:**
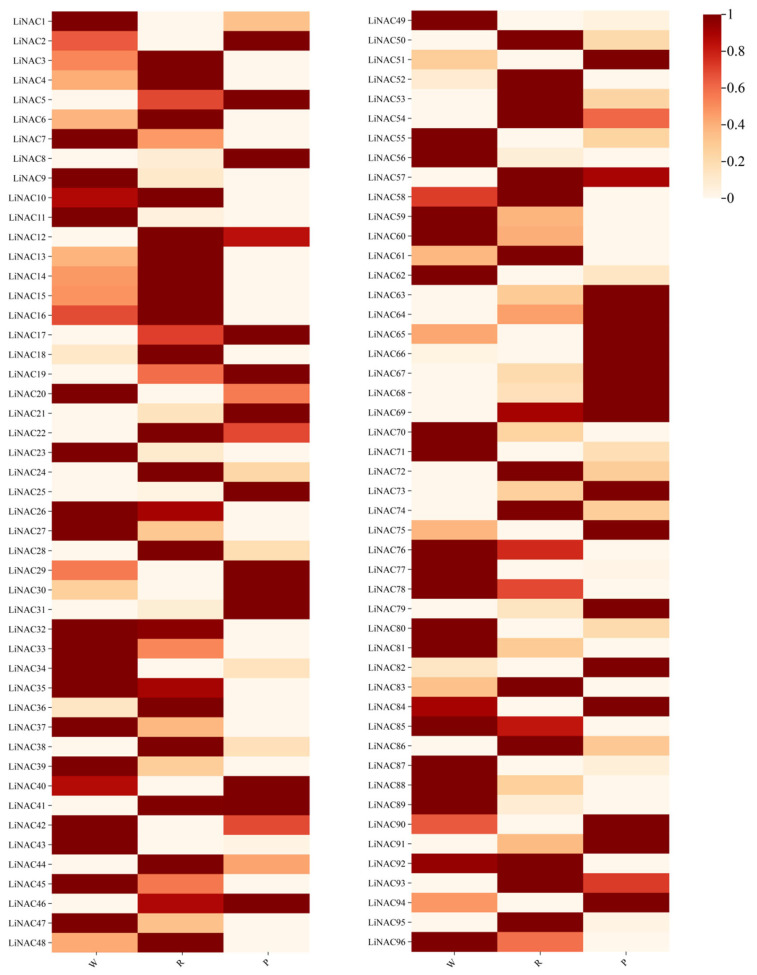
Heat map of expression levels of *LiNACs*. Three color varieties of *L. indica*—white (W), red (R), and purple (P).

Based on transcriptome data analysis, a total of 96 *LiNACs* were screened, and the screened *LiNACs* were subjected to heatmap plotting on Chiplot (Figure 10). Combining expression levels of anthocyanins screened from the metabolome (Supplementary Figure S1), 42 related *LiNACs* were selected, including 17 with the same and 25 with the opposite expression trend as anthocyanin content.

### 3.10. WGCNA of LiNACs

**Figure 11:**
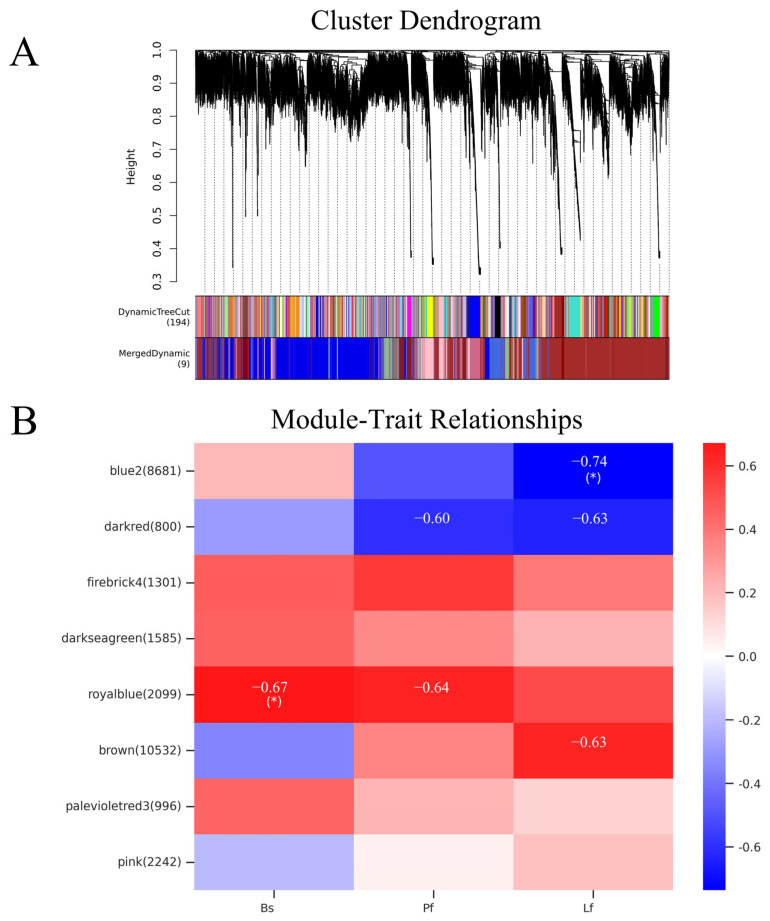
Weighted correlation network analysis. (**A**) Weighted correlation analysis and correlation module construction. (The upper part is the gene clustering tree prepared by constructing the dissTOM matrix based on the weighted correlation coefficients. The lower part of the figure shows the distribution of genes in each module. The same color represents the same module. The Dynamic Tree Cut color module was identified using the dynamic Tree Cut method, and the Merged Dynamic was the final module obtained. These modules were used for subsequent analysis. Different colors represent different modules.) (**B**) Characteristic module correlation heatmap. Statistical significance was determined using Student’s t-test (* *p* < 0.05).

**Figure 12:**
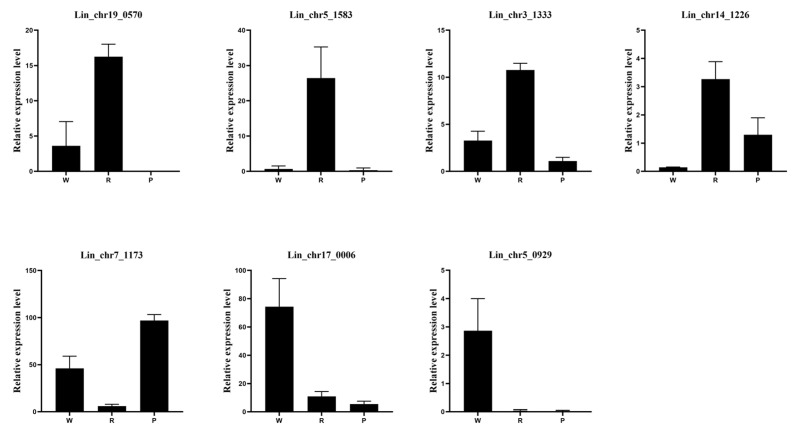
qRT-PCR analysis of seven *NAC* genes in *Lagerstroemia indica*, with the horizontal axis representing petals of different colors and the vertical axis representing relative expression levels.

We used the WGCNA to identify the *LiNACs* associated with the color of *L. indica* and selected nine modules labeled with different colors (Figure 11A). The number of genes in these modules ranged from 131 to 10,532 (Supplementary Table S4). By screening the modules co-expressed with the anthocyanin content of the petals during the buds (Bs), peak flowering (Pf), and late florescence (Lf) stages of *L. indica* (Figure 11B), three modules showed a positive correlation, while one showed a negative correlation. Both positive and negative modules contained significantly correlated modules, indicating that they might play an important role in anthocyanin synthesis in *L. indica* petals. Therefore, we selected the darkred and royalblue modules with significant correlation for subsequent analysis and identified three and four key *NACs*, respectively. Subsequently, qRT-PCR was conducted to verify the expression of these seven *NACs* genes. And the qRT-PCR results were consistent with the RNA-seq data (Figure 12).

### 3.11. Gene Co-Expression Network Analysis and Protein Interaction Network Analysis

**Figure 13:**
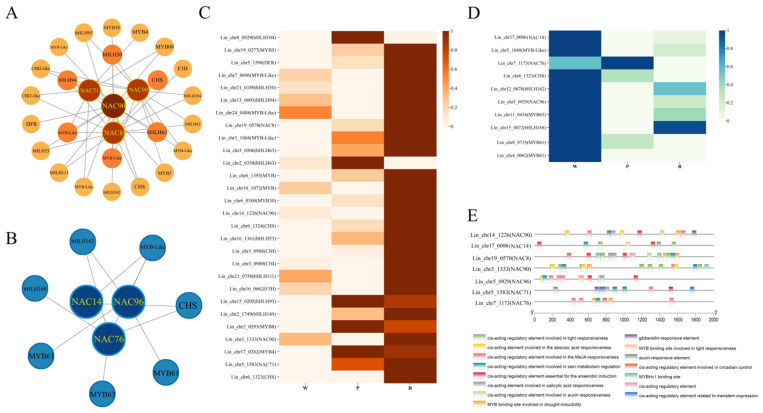
Construction of gene co-expression network and analysis of the cis-acting elements in the promoters of *LiNACs*. (**A**,**B**). The co-expression network diagrams of *NAC* and its co-expressed genes, with darker colors indicating more co-expressed genes. (**C**,**D**). Heatmaps of the expression levels of *NACs* and their co-expressed genes in the white (W), pink (P), and red (R) petals of crape myrtle. (**E**). The homologous element diagram of the first 2000 bp promoter region of *NAC*.

The seven *LiNACs* identified from Section 3.10 were used to construct gene co-expression networks using Cytoscape, resulting in two co-expression networks: The positively and negatively correlated co-expression networks (red and blue, respectively). Swiss Prot annotation of genes in the network showed that both networks might harbor TFs and genes related to anthocyanin expression. In the positively correlated co-expression network (Figure 13A), four *NACs* and anthocyanin related genes (four biosynthesis genes *DFR*, *F3H*, *CHI*, and *CHS*, and 18 *MYB* and *bHLH* TFs) were identified. The expression heatmap (Figure 13C) showed that except for *NAC90*, which had a higher expression level in white flowers than in red flowers, the expression levels of the other 27 genes were higher in both red flowers and pink flowers than in white flowers. Among them, the expression pattern of 22 genes was consistent with that of anthocyanin content changes.

In the negatively correlated co-expression network (Figure 13B), three *NACs*, one gene related to anthocyanin synthesis pathway regulation (*CHS*), and six TFs (*MYB* and *bHLH*) were identified. The expression heatmap (Figure 13D) showed that except for *bHLH168*, which had a higher expression level in pink flowers, the expression levels of the other nine genes were the highest in white flowers. Among them, the expression patterns of *MYB61* and *CHS* were completely opposite to the anthocyanin content changes.

**Figure 14:**
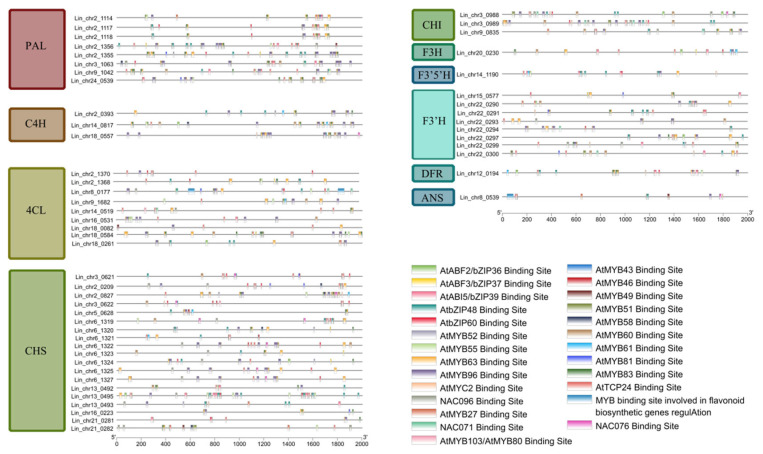
Analysis of homeotropic elements in the promoters of the anthocyanin-related genes in *Lagerstroemia indica*.

By screening the promoter cis-acting elements of *NACs* in the two co-expression networks, 15 promoter elements were identified (Figure 13E). These comprised homeoregulatory elements related to light response, MeJA reaction, maize protein metabolism regulation, anaerobic induction, meristematic tissue expression, and circadian rhythm control, cis-regulatory elements involved in ABA reaction, cis-acting elements involved in salicylic acid reactivity and auxin response, MYB binding sites involved in drought induction and light response, MYBHv1 binding sites, gibberellin response elements, and auxin response elements. Meanwhile, by screening the cis-acting elements of the promoters of genes related to the anthocyanin synthesis pathway (Figure 14), a total of 26 promoter elements were identified. Many protein binding sites (such as bZIP, MYB, NAC, TCP, and MYC) were found in these promoter elements. The MYB binding sites were related to flavonoid regulation in *4CL*, *CHS*, and *ANS*.

**Figure 15:**
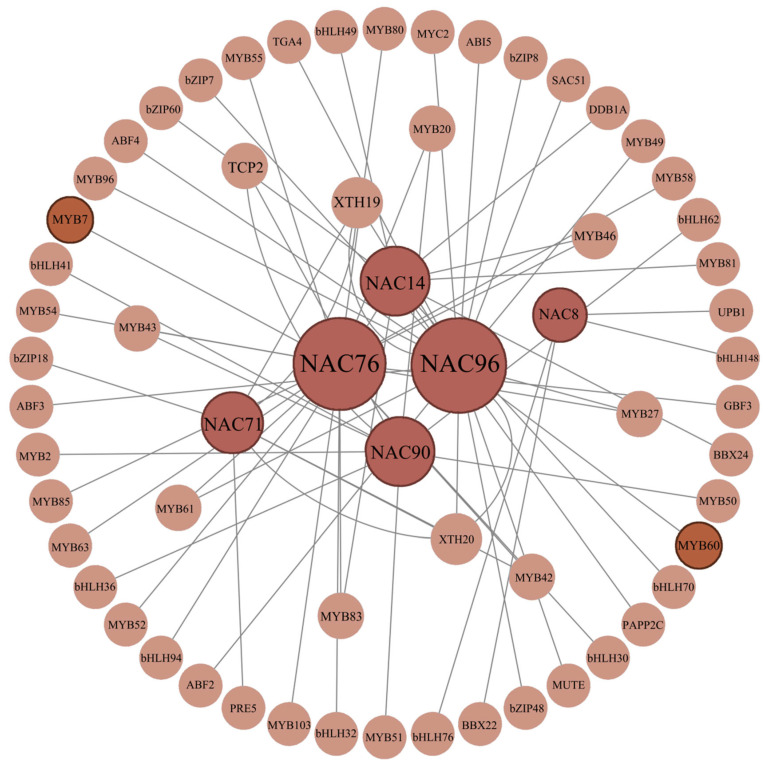
Interaction network of LiNAC proteins.

To further investigate the role of these *LiNACs* in anthocyanin metabolism, we performed an analysis of NAC-interacting proteins. A total of 62 proteins were screened in the protein–protein interaction network (Figure 15), including six NAC proteins, 23 MYB TFs, 10 bHLH TFs, five bZIP TFs, and 23 other proteins (including ABF, ABI, BBX, DDB1A, GBF, MUTE, MYC, PAPP2C, PRE, SAC, TCP, TGA, UPB, and XTH). NAC8, NAC10, NAC71, NAC76, NAC90, and NAC96 interact with 4, 10, 7, 19, 20, and 9 proteins, respectively (Figure 15). Among these, two proteins (MYB7 [31] and MYB60 [32]) related to anthocyanin synthesis regulation were screened out. The protein–protein interaction network analysis suggested potential interactions between NAC76 and MYB7, as well as NAC96 and MYB60, implying that NAC76 and NAC96 may participate in anthocyanin metabolism by interacting with MYB7 and MYB60, respectively.

## 4. Discussion

As a traditional Chinese plant, *L. indica* has enormous economic value in terms of ecological, medicinal, and ornamental purposes [33]. Flower color is one of the most important ornamental characteristics of *L. indica*, yet the regulatory mechanisms underlying its formation require further elucidation [34]. Plant color is determined by the pigment components in the petal cells and is influenced by various factors, including temperature and pH [35]. Petal color is mainly formed by flavonoids, carotenoids, and beet pigments [36]. Flavonoids are the largest and most widely present pigment group in plants and are crucial for the formation of flower colors in most plant species [37]. Most flavonoids are anthocyanins, which provide plants with different color schemes, such as red, purple, and blue [38]. Studies have shown that anthocyanins are the main pigments responsible for *L. indica* color [34]. Previous studies have explored the anthocyanin synthesis pathway in *L. indica* [30]; however, the regulation of anthocyanin synthesis in *L. indica* still needs to be elucidated.

The *NAC* family is one of the most important TF families in plants, and several studies have shown that it plays an important role in many growth, development, and flavonoid regulation processes in plants [39]. Studies showed that three *LiNACs* genes, *LiNAC2/8/13*, might regulate weeping traits in *L. indica* [40]. In addition, this family has been implicated in regulating plant growth, development, and environmental stress responses [4]. For instance, ANAC092/ORE1 inhibit the transcriptional activity of *GLKs*, leading to the occurrence and progression of leaf senescence [41]. ANAC019 and MYC2 form a complex that promotes the expression of certain *CCGs* in response to jasmonic acid (JA). This finding indicated that JA induces grading and coordinated regulation of MYC- and ANAC-mediated chlorophyll degradation [42]. Overexpression of the stress-inducible *SNAC1* gene in rice enhances drought and salt tolerance during the nutritional period [43].

Some studies have shown that *NAC* also regulates the anthocyanin synthesis pathways in plants [44]. For example, in Arabidopsis, *ANAC078* serves as a regulatory factor for anthocyanin biosynthesis. Under normal circumstances, ANAC078 protein expression is dormant. However, it activates and positively regulates anthocyanin synthesis-related genes (*DFR* and *LDOX*) under high light stress [45]. *ANAC032* overexpression inhibits anthocyanin accumulation and alters anthocyanin biosynthesis and regulatory gene expression under stress [46]. ABA induces interaction between MdNAC1 and bZIP TF, leading to anthocyanin synthesis in red-fleshed apples [47]. MdNAC14-Like inhibits anthocyanin synthesis by suppressing the transcriptional activities of *MdMYB9*, *MdMYB10*, and *MdUFGT* [48]. PaNAC03 has been found to act as an independent negative regulator in *Norway spruce*, acting as a repressor of the flavonoid synthesis pathway by interacting with the promoters of the genes related to this pathway (*CHS* and *F3′H*) [49]. *BoNAC019* overexpression in *Brassica oleracea* leads to decreased drought resistance, reactive oxygen species (ROS) levels, and anthocyanin accumulation [50].

We identified a total of 167 *NACs* in *L. indica*. These *NACs* were consistent with their counterparts in Arabidopsis, rice, *Populus tomentosa*, tea tree, etc. The NAM sequence in the NAC domain of *L. indica* was highly conserved (Figure 2). Through phylogenetic analysis, the identified *NACs* were divided into ten subgroups (Figure 6). The collinearity analysis identified 85 pairs of homologous genes in *L. indica* and *A. thaliana* (Figure 8). Gene duplication is key for expansion of the number of genes in a gene family [4]. The results suggested that a large number of gene replication events occurred, ranging from 106 *NACs* in *A. thaliana* to 167 *NACs* in *L. indica*. In order to explore the relationship between the detected *LiNACs* and the color of *L. indica*, we selected three different-colored ‘Jianmin Hong’ variety plants for further study and identified 96 *LiNACs* in the transcript annotation (Figure 10).

Two highly significant modules co-expressed with genes related to anthocyanin synthesis were screened using WGCNA, and seven *LiNACs* (four positively correlated and three negatively correlated) were annotated. The co-expression network analysis revealed that these *NACs* correlated with anthocyanin synthesis-related (*CHS*) and transcriptional regulatory genes (*MYB* and *bHLH*) (Figure 13A,B). Further heat map analysis revealed that the expression patterns of these genes were almost consistent (Figure 13C,D), indicating that these seven *NACs* might be related to anthocyanin synthesis. Due to the lack of transgenic and gene editing systems, this study mainly focuses on bioinformatics analysis and expression identification, and cannot verify the original function of genes in *L. indica*. Pending the establishment of transgenic and gene editing systems, further validation of the functions of sevens *NACs* is needed.

Many studies have shown that anthocyanin synthesis is regulated by MYB, bHLH, and WD40 TFs [51]. For example, PavMYB10.1 in cherry (*Prunus avium*) participates in anthocyanin biosynthesis and regulates fruit skin color [52]. The anthocyanin biosynthesis-related genes in *A. thaliana* are jointly regulated by MBW complex [53]. The loss of bHLH expression in carnations inhibits the synthesis of *DFR* and downstream anthocyanins, leading to reduced anthocyanin accumulation and whitening of petals [54].

Analysis of the cis-acting elements in the promoters of genes related to the anthocyanin biosynthesis pathway in *L. indica* showed that the *4CL*, *CHS*, and *ANS* promoters had MYB binding sites that regulate the flavonoid biosynthesis pathway. Evidence indicates that the architecture of genomic regulatory networks (GRNs) integrates multiple interaction modalities, spanning protein–protein binding, transcription factor-DNA binding, and cis/trans gene regulatory mechanisms [55]. The protein interaction network analysis revealed that many MYB proteins had interactive relationships (Figure 15) with two genes related to anthocyanin regulation (MYB7 [31] and MYB60 [32]). These findings showed that *LiNACs* might participate in the regulation of anthocyanin synthesis by interacting with MYB7 or MYB60 proteins. However, it warrants further elucidation. Meanwhile, the binding sites of NAC71, NAC76, and NAC96 were identified in the promoter element analysis of genes related to the anthocyanin synthesis pathway, indicating that they might also directly regulate anthocyanin synthesis through binding sites. However, in *A. thaliana*, these *NAC* homologs have not been reported to participate in anthocyanin metabolism. Studies demonstrate that these *Arabidopsis NAC* genes are primarily involved in plant immunity, tissue reunion, and growth arrest, among other biological processes [56,57,58]

We also discovered other related proteins (ABF, ABI, bZIP, MYB, and MYC) that interacted with LiNAC. These proteins harbored binding sites on the genes related to the anthocyanin synthesis pathway. Hence, we speculated that LiNAC might indirectly affect anthocyanin synthesis by interacting with these proteins. Since *L. indica* does not have a protein database, we used *A. thaliana* database (STRING database) for the interaction relationship between homologous proteins. Due to the differences between *A. thaliana* and *L. indica* species, the complete interaction relationship of LiNAC protein in *L. indica* cannot be fully captured. The predicted protein interaction relationship needs to be further verified by experiments.

In addition, we found several plant hormone-binding elements (such as auxin, gibberellin, and MeJA) in the upstream homeotropic expression elements of seven *LiNACs*. Studies have shown a regulatory relationship between auxin and anthocyanin synthesis. For instance, in red raspberry (*Rubus idaeus* L.). auxin reverse regulates anthocyanin metabolism [59]. In *A. thaliana*, gibberellin negatively regulates flavonoid biosynthesis by reducing *GA4* expression, promoting anthocyanin accumulation [60]. Exogenous addition of auxin promotes anthocyanin synthesis in non-calcareous sweet cherries (*Prunus avium* L.) [61]. MeJA promotes anthocyanin accumulation in ‘Fuji’ apples and alters the production of phenols and pigments [62] In Caitai, MeJA-induced bHLH42 mediates tissue-specific accumulation of anthocyanins by regulating flavonoid metabolism-related pathways [63]. Therefore, the regulatory elements of auxin, gibberellin, and MeJA were found during promoter element detection of *LiNACs*, indicating that *LiNACs* might regulate the anthocyanin pathway via the plant hormones (auxin, gibberellin, and MeJA).

We also found photoresponsive elements while screening the promoters of the seven *LiNACs*, indicating that these *LiNACs* can respond to light. Studies have shown that the synthesis of plant anthocyanins is regulated by light. For instance, natural UV intensity and high temperature promote the acylation of anthocyanins (derivatives of delphinidin and petunian) in grapes [64]. During the development of grape berries, processing light quality/quantity (UVB) and temperature can also affect the accumulation of flavonols and anthocyanins [65]. The light-responsive TF PpWRKY44 positively regulates light-induced anthocyanin biosynthesis by directly activating the PpMYB10 promoter in red pear fruit [66]. Therefore, we speculated that these LiNACs might participate in light-induced regulation of anthocyanin synthesis by responding to light reactions.

## 5. Conclusions

This study is a comprehensive analysis of the *NAC* gene family based on the *L. indica* genome. A total of 167 *LiNACs* were screened from the *L. indica* genome. These genes were distributed across 23 chromosomes and harbored conserved NAM sequences. Based on their phylogenetic tree, the genes were divided into ten subgroups. Transcriptome data analysis led to the identification of 96 *LiNACs*. WGCNA analysis and expression heatmap analysis revealed seven *LiNACs* associated with anthocyanin synthesis. Promoter analysis and protein interaction analysis indicated that *LiNACs* might regulate anthocyanin synthesis through protein interactions with MYB, ABF, ABI, bZIP, MYC, and other proteins. In addition, *LiNACs* might also be involved in the regulation of anthocyanin synthesis pathways through plant hormones and light reactions. Our results could prove valuable for the functional identification of *LiNACs* and the genetic improvement of *L. indica*.

## Data Availability

Data are contained within the article.

## Supplementary Materials

The following supporting information can be downloaded at: https://www.mdpi.com/article/10.3390/cimb47070542/s1.
