# Supplementary figures and images for: NAC Gene Family in Lagerstroemia indica: Genome-Wide Identification, Characterization, Expression Analysis, and Key Regulators Involved in Anthocyanin Biosynthesis
Source: Curr Issues Mol Biol. 2025 Jul 11;47(7):542. doi: 10.3390/cimb47070542 (PMC12293354; doi:10.3390/cimb47070542)

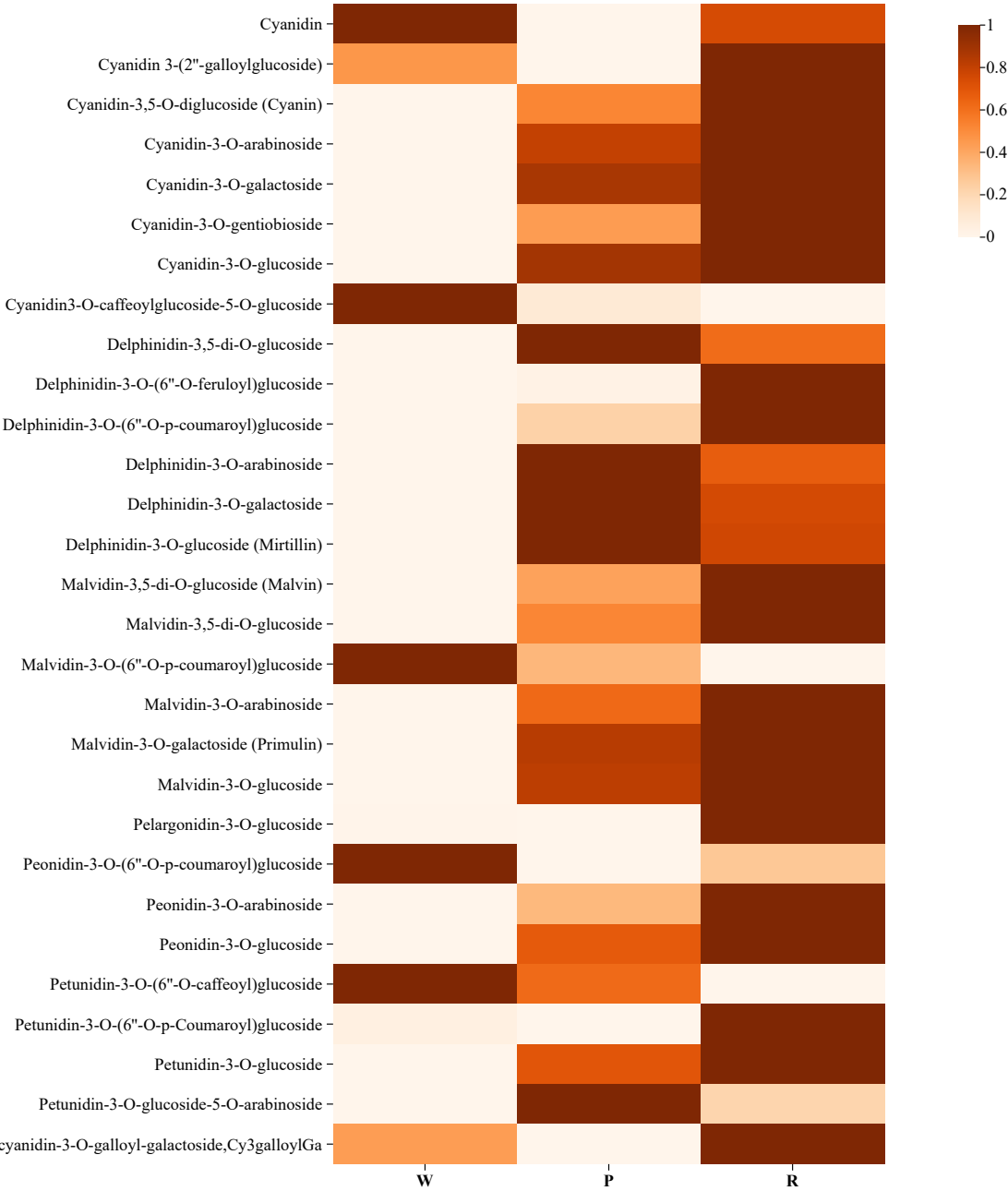

Supplement: Supplementary file 1 [file cimb-47-00542-s001.zip › Figure S1. Thermogram of anthocyanin metabolites in the three-color varieties (white, red, and purple) of L. indica.pdf]
